# Supplementary material for: Low to moderate wave exposure did not impact blue mussel (Mytilus edulis) growth in a mesocosm study
Source: PLoS One. 2024 Dec 5;19(12):e0315136. doi: 10.1371/journal.pone.0315136 (PMC11620627; doi:10.1371/journal.pone.0315136)
Supplement: S1 Table — BN = basin number; MN = mussel number; WE = wave exposure level; L = length (mm); LC = change in length from one week to the other, for each mussel in each basin; WI = width (mm); WIC = Width change; T = thickness (mm); TC = thickness change; WE = weight (mm); WEC = weight change; DV = displacement volume; DVC = displacement volume change. (DOCX) [file pone.0315136.s001.docx]

**S1 Table.** Data on length, width, thickness, weight and displacement volume of blue mussel (Mytilus edulis) measured weekly in a mesocosm study over 13 weeks in 2022. BN = basin number; MN = mussel number; WE = wave exposure level; L = length (mm); LC = change in length from one week to the other, for each mussel in each basin; WI = width (mm); WIC = Width change; T = thickness (mm); TC = thickness change; WE = weight (mm); WEC = weight change; DV = displacement volume; DVC = displacement volume change.

| **Date** | **BN** | **MN** | **WE** | **Length (mm)** | | **Width (mm)** | | **Thicknes**  **(mm)** | | **Weight**  **(g)** | | **Displacement**  **volume** | |
| --- | --- | --- | --- | --- | --- | --- | --- | --- | --- | --- | --- | --- | --- |
|  |  |  |  | **L** | **LC** | **WI** | **WIC** | **T** | **TC** | **WE** | **WEC** | **DV** | **DVC** |
| 22.06 | 1 | 1 | High | 29 | 0.00 | 18 | 0.00 | 12 | 0.00 | 3.04 | 0.00 | 3 | 0.00 |
| 22.06 | 1 | 2 | High | 29 | 0.00 | 16 | 0.00 | 9 | 0.00 | 2.89 | 0.00 | 2 | 0.00 |
| 22.06 | 1 | 3 | High | 28 | 0.00 | 17 | 0.00 | 11 | 0.00 | 3.50 | 0.00 | 3 | 0.00 |
| 22.06 | 2 | 4 | High | 28 | 0.00 | 14 | 0.00 | 10 | 0.00 | 3.03 | 0.00 | 3 | 0.00 |
| 22.06 | 2 | 5 | High | 30 | 0.00 | 16 | 0.00 | 10 | 0.00 | 3.37 | 0.00 | 3 | 0.00 |
| 22.06 | 2 | 6 | High | 29 | 0.00 | 16 | 0.00 | 11 | 0.00 | 3.54 | 0.00 | 3 | 0.00 |
| 22.06 | 3 | 7 | Low | 29 | 0.00 | 15 | 0.00 | 11 | 0.00 | 3.32 | 0.00 | 3 | 0.00 |
| 22.06 | 3 | 8 | Low | 32 | 0.00 | 16 | 0.00 | 12 | 0.00 | 4.36 | 0.00 | 3 | 0.00 |
| 22.06 | 3 | 9 | Low | 29 | 0.00 | 15 | 0.00 | 10 | 0.00 | 3.13 | 0.00 | 2 | 0.00 |
| 22.06 | 4 | 10 | Low | 30 | 0.00 | 16 | 0.00 | 10 | 0.00 | 3.23 | 0.00 | 3 | 0.00 |
| 22.06 | 4 | 11 | Low | 28 | 0.00 | 14 | 0.00 | 10 | 0.00 | 3.25 | 0.00 | 2 | 0.00 |
| 22.06 | 4 | 12 | Low | 29 | 0.00 | 16 | 0.00 | 9 | 0.00 | 3.35 | 0.00 | 3 | 0.00 |
| 22.06 | 5 | 13 | Low | 27 | 0.00 | 14 | 0.00 | 9 | 0.00 | 2.72 | 0.00 | 3 | 0.00 |
| 22.06 | 5 | 14 | Low | 27 | 0.00 | 14 | 0.00 | 9 | 0.00 | 2.89 | 0.00 | 2 | 0.00 |
| 22.06 | 5 | 15 | Low | 29 | 0.00 | 16 | 0.00 | 10 | 0.00 | 3.75 | 0.00 | 3 | 0.00 |
| 22.06 | 6 | 16 | Low | 30 | 0.00 | 16 | 0.00 | 10 | 0.00 | 3.30 | 0.00 | 3 | 0.00 |
| 22.06 | 6 | 17 | Low | 29 | 0.00 | 14 | 0.00 | 9 | 0.00 | 3.01 | 0.00 | 3 | 0.00 |
| 22.06 | 6 | 18 | Low | 28 | 0.00 | 15 | 0.00 | 11 | 0.00 | 3.53 | 0.00 | 2 | 0.00 |
| 22.06 | 7 | 19 | High | 29 | 0.00 | 16 | 0.00 | 11 | 0.00 | 3.59 | 0.00 | 3 | 0.00 |
| 22.06 | 7 | 20 | High | 28 | 0.00 | 16 | 0.00 | 10 | 0.00 | 3.23 | 0.00 | 2 | 0.00 |
| 22.06 | 7 | 21 | High | 28 | 0.00 | 15 | 0.00 | 10 | 0.00 | 3.32 | 0.00 | 3 | 0.00 |
| 22.06 | 8 | 22 | High | 31 | 0.00 | 16 | 0.00 | 10 | 0.00 | 3.41 | 0.00 | 3 | 0.00 |
| 22.06 | 8 | 23 | High | 28 | 0.00 | 15 | 0.00 | 10 | 0.00 | 2.91 | 0.00 | 3 | 0.00 |
| 22.06 | 8 | 24 | High | 31 | 0.00 | 16 | 0.00 | 11 | 0.00 | 3.55 | 0.00 | 3 | 0.00 |
| 22.06 | 9 | 25 | Low | 28 | 0.00 | 18 | 0.00 | 10 | 0.00 | 2.84 | 0.00 | 3 | 0.00 |
| 22.06 | 9 | 26 | Low | 30 | 0.00 | 15 | 0.00 | 9 | 0.00 | 3.20 | 0.00 | 3 | 0.00 |
| 22.06 | 9 | 27 | Low | 29 | 0.00 | 14 | 0.00 | 12 | 0.00 | 3.45 | 0.00 | 3 | 0.00 |
| 22.06 | 10 | 28 | Low | 27 | 0.00 | 14 | 0.00 | 8 | 0.00 | 2.28 | 0.00 | 2 | 0.00 |
| 22.06 | 10 | 29 | Low | 29 | 0.00 | 14 | 0.00 | 10 | 0.00 | 3.05 | 0.00 | 3 | 0.00 |
| 22.06 | 10 | 30 | Low | 28 | 0.00 | 13 | 0.00 | 10 | 0.00 | 2.80 | 0.00 | 3 | 0.00 |
| 22.06 | 11 | 31 | High | 28 | 0.00 | 16 | 0.00 | 9 | 0.00 | 2.88 | 0.00 | 2 | 0.00 |
| 22.06 | 11 | 32 | High | 27 | 0.00 | 16 | 0.00 | 9 | 0.00 | 2.70 | 0.00 | 3 | 0.00 |
| 22.06 | 11 | 33 | High | 27 | 0.00 | 15 | 0.00 | 9 | 0.00 | 2.67 | 0.00 | 2 | 0.00 |
| 22.06 | 12 | 34 | High | 28 | 0.00 | 15 | 0.00 | 10 | 0.00 | 3.21 | 0.00 | 3 | 0.00 |
| 22.06 | 12 | 35 | High | 30 | 0.00 | 15 | 0.00 | 10 | 0.00 | 3.29 | 0.00 | 3 | 0.00 |
| 22.06 | 12 | 36 | High | 28 | 0.00 | 15 | 0.00 | 10 | 0.00 | 3.19 | 0.00 | 3 | 0.00 |
| 28.06 | 1 | 1 | High | 31 | 6.45 | 18 | 0.00 | 11 | -9.09 | 4.03 | 24.57 | 4 | 25.00 |
| 28.06 | 1 | 2 | High | 30 | 3.33 | 17 | 5.88 | 10 | 10.00 | 3.46 | 16.47 | 2 | 0.00 |
| 28.06 | 1 | 3 | High | 30 | 6.67 | 16 | -6.25 | 10 | -10.00 | 3.63 | 3.58 | 3 | 0.00 |
| 28.06 | 2 | 4 | High | 29 | 3.45 | 15 | 6.67 | 10 | 0.00 | 3.10 | 2.26 | 3 | 0.00 |
| 28.06 | 2 | 5 | High | 31 | 3.23 | 17 | 5.88 | 11 | 9.09 | 3.45 | 2.32 | 3 | 0.00 |
| 28.06 | 2 | 6 | High | 30 | 3.33 | 17 | 5.88 | 11 | 0.00 | 3.71 | 4.58 | 3 | 0.00 |
| 28.06 | 3 | 7 | Low | 30 | 3.33 | 15 | 0.00 | 11 | 0.00 | 3.34 | 0.60 | 3 | 0.00 |
| 28.06 | 3 | 8 | Low | 32 | 0.00 | 16 | 0.00 | 12 | 0.00 | 4.26 | -2.35 | 4 | 25.00 |
| 28.06 | 3 | 9 | Low | 30 | 3.33 | 16 | 6.25 | 11 | 9.09 | 3.24 | 3.40 | 3 | 33.33 |
| 28.06 | 4 | 10 | Low | 31 | 3.23 | 16 | 0.00 | 10 | 0.00 | 3.20 | -0.94 | 3 | 0.00 |
| 28.06 | 4 | 11 | Low | 29 | 3.45 | 15 | 6.67 | 11 | 9.09 | 3.27 | 0.61 | 3 | 33.33 |
| 28.06 | 4 | 12 | Low | 30 | 3.33 | 16 | 0.00 | 9 | 0.00 | 3.37 | 0.59 | 3 | 0.00 |
| 28.06 | 5 | 13 | Low | 28 | 3.57 | 14 | 0.00 | 9 | 0.00 | 2.75 | 1.09 | 3 | 0.00 |
| 28.06 | 5 | 14 | Low | 28 | 3.57 | 14 | 0.00 | 10 | 10.00 | 2.80 | -3.21 | 2 | 0.00 |
| 28.06 | 5 | 15 | Low | 30 | 3.33 | 16 | 0.00 | 10 | 0.00 | 3.71 | -1.08 | 2 | -50.00 |
| 28.06 | 6 | 16 | Low | 29 | -3.45 | 15 | -6.67 | 10 | 0.00 | 3.32 | 0.60 | 3 | 0.00 |
| 28.06 | 6 | 17 | Low | 29 | 0.00 | 14 | 0.00 | 9 | 0.00 | 2.70 | -11.48 | 2 | -50.00 |
| 28.06 | 6 | 18 | Low | 29 | 3.45 | 15 | 0.00 | 10 | -10.00 | 3.34 | -5.69 | 3 | 33.33 |
| 28.06 | 7 | 19 | High | 29 | 0.00 | 16 | 0.00 | 11 | 0.00 | 3.32 | -8.13 | 3 | 0.00 |
| 28.06 | 7 | 20 | High | 29 | 3.45 | 16 | 0.00 | 10 | 0.00 | 3.19 | -1.25 | 3 | 33.33 |
| 28.06 | 7 | 21 | High | 29 | 3.45 | 15 | 0.00 | 11 | 9.09 | 3.36 | 1.19 | 3 | 0.00 |
| 28.06 | 8 | 22 | High | 31 | 0.00 | 16 | 0.00 | 10 | 0.00 | 3.45 | 1.16 | 3 | 0.00 |
| 28.06 | 8 | 23 | High | 28 | 0.00 | 15 | 0.00 | 10 | 0.00 | 2.70 | -7.78 | 3 | 0.00 |
| 28.06 | 8 | 24 | High | 31 | 0.00 | 16 | 0.00 | 11 | 0.00 | 3.67 | 3.27 | 3 | 0.00 |
| 28.06 | 9 | 25 | Low | 28 | 0.00 | 15 | -20.00 | 10 | 0.00 | 2.95 | 3.73 | 3 | 0.00 |
| 28.06 | 9 | 26 | Low | 31 | 3.23 | 15 | 0.00 | 10 | 10.00 | 3.01 | -6.31 | 3 | 0.00 |
| 28.06 | 9 | 27 | Low | 29 | 0.00 | 15 | 6.67 | 13 | 7.69 | 3.48 | 0.86 | 3 | 0.00 |
| 28.06 | 10 | 28 | Low | 27 | 0.00 | 14 | 0.00 | 8 | 0.00 | 2.37 | 3.80 | 2 | 0.00 |
| 28.06 | 10 | 29 | Low | 29 | 0.00 | 14 | 0.00 | 10 | 0.00 | 3.01 | -1.33 | 3 | 0.00 |
| 28.06 | 10 | 30 | Low | 28 | 0.00 | 14 | 7.14 | 10 | 0.00 | 2.83 | 1.06 | 2 | -50.00 |
| 28.06 | 11 | 31 | High | 29 | 3.45 | 15 | -6.67 | 9 | 0.00 | 2.79 | -3.23 | 3 | 33.33 |
| 28.06 | 11 | 32 | High | 29 | 6.90 | 15 | -6.67 | 9 | 0.00 | 2.78 | 2.88 | 3 | 0.00 |
| 28.06 | 11 | 33 | High | 27 | 0.00 | 14 | -7.14 | 9 | 0.00 | 2.65 | -0.75 | 2 | 0.00 |
| 28.06 | 12 | 34 | High | 29 | 3.45 | 15 | 0.00 | 10 | 0.00 | 3.25 | 1.23 | 3 | 0.00 |
| 28.06 | 12 | 35 | High | 31 | 3.23 | 15 | 0.00 | 10 | 0.00 | 3.18 | -3.46 | 3 | 0.00 |
| 28.06 | 12 | 36 | High | 28 | 0.00 | 15 | 0.00 | 10 | 0.00 | 3.21 | 0.62 | 3 | 0.00 |
| 05.07 | 1 | 1 | High | 31 | 0.00 | 17 | -5.88 | 12 | 8.33 | 4.12 | 2.18 | 3 | -33.33 |
| 05.07 | 1 | 2 | High | 30 | 0.00 | 17 | 0.00 | 10 | 0.00 | 3.34 | -3.59 | 3 | 33.33 |
| 05.07 | 1 | 3 | High | 29 | -3.45 | 17 | 5.88 | 11 | 9.09 | 3.77 | 3.71 | 3 | 0.00 |
| 05.07 | 2 | 4 | High | 29 | 0.00 | 15 | 0.00 | 10 | 0.00 | 3.20 | 3.13 | 3 | 0.00 |
| 05.07 | 2 | 5 | High | 31 | 0.00 | 16 | -6.25 | 11 | 0.00 | 3.65 | 5.48 | 3 | 0.00 |
| 05.07 | 2 | 6 | High | 30 | 0.00 | 17 | 0.00 | 11 | 0.00 | 3.99 | 7.02 | 4 | 25.00 |
| 05.07 | 3 | 7 | Low | 29 | -3.45 | 15 | 0.00 | 11 | 0.00 | 3.43 | 2.62 | 3 | 0.00 |
| 05.07 | 3 | 8 | Low | 32 | 0.00 | 16 | 0.00 | 12 | 0.00 | 3.35 | -27.16 | 4 | 0.00 |
| 05.07 | 3 | 9 | Low | 31 | 3.23 | 16 | 0.00 | 11 | 0.00 | 3.60 | 10.00 | 3 | 0.00 |
| 05.07 | 4 | 10 | Low | 31 | 0.00 | 15 | -6.67 | 10 | 0.00 | 3.40 | 5.88 | 3 | 0.00 |
| 05.07 | 4 | 11 | Low | 30 | 3.33 | 15 | 0.00 | 11 | 0.00 | 3.49 | 6.30 | 3 | 0.00 |
| 05.07 | 4 | 12 | Low | 31 | 3.23 | 16 | 0.00 | 10 | 10.00 | 3.61 | 6.65 | 3 | 0.00 |
| 05.07 | 5 | 13 | Low | 28 | 0.00 | 15 | 6.67 | 10 | 10.00 | 2.99 | 8.03 | 3 | 0.00 |
| 05.07 | 5 | 14 | Low | 29 | 3.45 | 15 | 6.67 | 10 | 0.00 | 3.03 | 7.59 | 2 | 0.00 |
| 05.07 | 5 | 15 | Low | 30 | 0.00 | 16 | 0.00 | 10 | 0.00 | 3.70 | -0.27 | 3 | 33.33 |
| 05.07 | 6 | 16 | Low | 30 | 3.33 | 16 | 6.25 | 10 | 0.00 | 3.39 | 2.06 | 3 | 0.00 |
| 05.07 | 6 | 17 | Low | 30 | 3.33 | 15 | 6.67 | 10 | 10.00 | 2.91 | 7.22 | 3 | 33.33 |
| 05.07 | 6 | 18 | Low | 30 | 3.33 | 15 | 0.00 | 11 | 9.09 | 3.64 | 8.24 | 3 | 0.00 |
| 05.07 | 7 | 19 | High | 29 | 0.00 | 16 | 0.00 | 12 | 8.33 | 3.59 | 7.52 | 3 | 0.00 |
| 05.07 | 7 | 20 | High | 30 | 3.33 | 16 | 0.00 | 10 | 0.00 | 3.30 | 3.33 | 2 | -50.00 |
| 05.07 | 7 | 21 | High | 29 | 0.00 | 15 | 0.00 | 11 | 0.00 | 3.48 | 3.45 | 3 | 0.00 |
| 05.07 | 8 | 22 | High | 32 | 3.13 | 17 | 5.88 | 11 | 9.09 | 3.55 | 2.82 | 3 | 0.00 |
| 05.07 | 8 | 23 | High | 29 | 3.45 | 16 | 6.25 | 10 | 0.00 | 3.24 | 16.67 | 3 | 0.00 |
| 05.07 | 8 | 24 | High | 32 | 3.13 | 16 | 0.00 | 11 | 0.00 | 3.99 | 8.02 | 3 | 0.00 |
| 05.07 | 9 | 25 | Low | 28 | 0.00 | 15 | 0.00 | 10 | 0.00 | 3.08 | 4.22 | 3 | 0.00 |
| 05.07 | 9 | 26 | Low | 31 | 0.00 | 16 | 6.25 | 10 | 0.00 | 3.51 | 14.25 | 3 | 0.00 |
| 05.07 | 9 | 27 | Low | 31 | 6.45 | 15 | 0.00 | 13 | 0.00 | 3.93 | 11.45 | 4 | 25.00 |
| 05.07 | 10 | 28 | Low | 28 | 3.57 | 14 | 0.00 | 8 | 0.00 | 2.49 | 4.82 | 3 | 33.33 |
| 05.07 | 10 | 29 | Low | 30 | 3.33 | 15 | 6.67 | 10 | 0.00 | 3.19 | 5.64 | 3 | 0.00 |
| 05.07 | 10 | 30 | Low | 29 | 3.45 | 14 | 0.00 | 11 | 9.09 | 2.96 | 4.39 | 3 | 33.33 |
| 05.07 | 11 | 31 | High | 29 | 0.00 | 15 | 0.00 | 9 | 0.00 | 3.07 | 9.12 | 3 | 0.00 |
| 05.07 | 11 | 32 | High | 28 | -3.57 | 15 | 0.00 | 9 | 0.00 | 3.03 | 8.25 | 3 | 0.00 |
| 05.07 | 11 | 33 | High | 27 | 0.00 | 14 | 0.00 | 10 | 10.00 | 2.80 | 5.36 | 2 | 0.00 |
| 05.07 | 12 | 34 | High | 29 | 0.00 | 16 | 6.25 | 11 | 9.09 | 3.48 | 6.61 | 3 | 0.00 |
| 05.07 | 12 | 35 | High | 31 | 0.00 | 16 | 6.25 | 10 | 0.00 | 3.45 | 7.83 | 3 | 0.00 |
| 05.07 | 12 | 36 | High | 28 | 0.00 | 16 | 6.25 | 11 | 9.09 | 3.40 | 5.59 | 3 | 0.00 |
| 12.07 | 1 | 1 | High | 31 | 0.00 | 18 | 5.56 | 12 | 0.00 | 4.21 | 2.14 | 4 | 25.00 |
| 12.07 | 1 | 2 | High | 31 | 3.23 | 17 | 0.00 | 10 | 0.00 | 3.79 | 11.87 | 3 | 0.00 |
| 12.07 | 1 | 3 | High | 30 | 3.33 | 17 | 0.00 | 11 | 0.00 | 3.90 | 3.33 | 3 | 0.00 |
| 12.07 | 2 | 4 | High | 29 | 0.00 | 15 | 0.00 | 10 | 0.00 | 3.31 | 3.32 | 3 | 0.00 |
| 12.07 | 2 | 5 | High | 31 | 0.00 | 17 | 5.88 | 11 | 0.00 | 3.77 | 3.18 | 3 | 0.00 |
| 12.07 | 2 | 6 | High | 30 | 0.00 | 18 | 5.56 | 11 | 0.00 | 4.17 | 4.32 | 3 | -33.33 |
| 12.07 | 3 | 7 | Low | 29 | 0.00 | 15 | 0.00 | 11 | 0.00 | 3.57 | 3.92 | 3 | 0.00 |
| 12.07 | 3 | 8 | Low | 32 | 0.00 | 16 | 0.00 | 12 | 0.00 | 4.41 | 24.04 | 4 | 0.00 |
| 12.07 | 3 | 9 | Low | 32 | 3.13 | 17 | 5.88 | 11 | 0.00 | 3.96 | 9.09 | 3 | 0.00 |
| 12.07 | 4 | 10 | Low | 31 | 0.00 | 16 | 6.25 | 10 | 0.00 | 3.62 | 6.08 | 3 | 0.00 |
| 12.07 | 4 | 11 | Low | 31 | 3.23 | 15 | 0.00 | 11 | 0.00 | 3.72 | 6.18 | 3 | 0.00 |
| 12.07 | 4 | 12 | Low | 31 | 0.00 | 17 | 5.88 | 10 | 0.00 | 3.77 | 4.24 | 3 | 0.00 |
| 12.07 | 5 | 13 | Low | 29 | 3.45 | 16 | 6.25 | 10 | 0.00 | 2.72 | -9.93 | 2 | -50.00 |
| 12.07 | 5 | 14 | Low | 30 | 3.33 | 15 | 0.00 | 10 | 0.00 | 3.29 | 7.90 | 3 | 33.33 |
| 12.07 | 5 | 15 | Low | 31 | 3.23 | 16 | 0.00 | 11 | 9.09 | 3.91 | 5.37 | 3 | 0.00 |
| 12.07 | 6 | 16 | Low | 30 | 0.00 | 16 | 0.00 | 10 | 0.00 | 3.49 | 2.87 | 3 | 0.00 |
| 12.07 | 6 | 17 | Low | 31 | 3.23 | 15 | 0.00 | 10 | 0.00 | 2.48 | -17.34 | 3 | 0.00 |
| 12.07 | 6 | 18 | Low | 31 | 3.23 | 16 | 6.25 | 11 | 0.00 | 3.90 | 6.67 | 4 | 25.00 |
| 12.07 | 7 | 19 | High | 30 | 3.33 | 16 | 0.00 | 12 | 0.00 | 3.68 | 2.45 | 3 | 0.00 |
| 12.07 | 7 | 20 | High | 30 | 0.00 | 17 | 5.88 | 10 | 0.00 | 3.53 | 6.52 | 3 | 33.33 |
| 12.07 | 7 | 21 | High | 30 | 3.33 | 15 | 0.00 | 11 | 0.00 | 3.55 | 1.97 | 3 | 0.00 |
| 12.07 | 8 | 22 | High | 33 | 3.03 | 17 | 0.00 | 10 | -10.00 | 3.97 | 10.58 | 4 | 25.00 |
| 12.07 | 8 | 23 | High | 30 | 3.33 | 16 | 0.00 | 10 | 0.00 | 3.52 | 7.95 | 3 | 0.00 |
| 12.07 | 8 | 24 | High | 32 | 0.00 | 16 | 0.00 | 11 | 0.00 | 4.06 | 1.72 | 3 | 0.00 |
| 12.07 | 9 | 25 | Low | 29 | 3.45 | 15 | 0.00 | 10 | 0.00 | 3.25 | 5.23 | 2 | -50.00 |
| 12.07 | 9 | 26 | Low | 32 | 3.13 | 16 | 0.00 | 10 | 0.00 | 3.70 | 5.14 | 3 | 0.00 |
| 12.07 | 9 | 27 | Low | 31 | 0.00 | 15 | 0.00 | 13 | 0.00 | 4.21 | 6.65 | 4 | 0.00 |
| 12.07 | 10 | 28 | Low | 29 | 3.45 | 15 | 6.67 | 9 | 11.11 | 2.76 | 9.78 | 2 | -50.00 |
| 12.07 | 10 | 29 | Low | 30 | 0.00 | 15 | 0.00 | 10 | 0.00 | 3.34 | 4.49 | 3 | 0.00 |
| 12.07 | 10 | 30 | Low | 29 | 0.00 | 14 | 0.00 | 11 | 0.00 | 2.85 | -3.86 | 3 | 0.00 |
| 12.07 | 11 | 31 | High | 29 | 0.00 | 16 | 6.25 | 10 | 10.00 | 3.30 | 6.97 | 2 | -50.00 |
| 12.07 | 11 | 32 | High | 29 | 3.45 | 16 | 6.25 | 10 | 10.00 | 3.23 | 6.19 | 3 | 0.00 |
| 12.07 | 11 | 33 | High | 28 | 3.57 | 15 | 6.67 | 10 | 0.00 | 3.06 | 8.50 | 3 | 33.33 |
| 12.07 | 12 | 34 | High | 30 | 3.33 | 16 | 0.00 | 11 | 0.00 | 3.59 | 3.06 | 3 | 0.00 |
| 12.07 | 12 | 35 | High | 32 | 3.13 | 16 | 0.00 | 10 | 0.00 | 3.64 | 5.22 | 3 | 0.00 |
| 12.07 | 12 | 36 | High | 29 | 3.45 | 16 | 0.00 | 11 | 0.00 | 3.54 | 3.95 | 3 | 0.00 |
| 18.07 | 1 | 1 | High | 31 | 0.00 | 18 | 0.00 | 12 | 0.00 | 4.29 | 1.86 | 3 | -33.33 |
| 18.07 | 1 | 2 | High | 32 | 3.13 | 17 | 0.00 | 11 | 9.09 | 3.93 | 3.56 | 3 | 0.00 |
| 18.07 | 1 | 3 | High | 30 | 0.00 | 17 | 0.00 | 11 | 0.00 | 3.97 | 1.76 | 4 | 25.00 |
| 18.07 | 2 | 4 | High | 29 | 0.00 | 15 | 0.00 | 10 | 0.00 | 2.70 | -22.59 | 3 | 0.00 |
| 18.07 | 2 | 5 | High | 32 | 3.13 | 16 | -6.25 | 11 | 0.00 | 3.96 | 4.80 | 3 | 0.00 |
| 18.07 | 2 | 6 | High | 31 | 3.23 | 17 | -5.88 | 11 | 0.00 | 4.34 | 3.92 | 4 | 25.00 |
| 18.07 | 3 | 7 | Low | 30 | 3.33 | 16 | 6.25 | 11 | 0.00 | 3.72 | 4.03 | 3 | 0.00 |
| 18.07 | 3 | 8 | Low | 33 | 3.03 | 16 | 0.00 | 12 | 0.00 | 4.41 | 0.00 | 4 | 0.00 |
| 18.07 | 3 | 9 | Low | 33 | 3.03 | 17 | 0.00 | 12 | 8.33 | 4.33 | 8.55 | 3 | 0.00 |
| 18.07 | 4 | 10 | Low | 32 | 3.13 | 16 | 0.00 | 10 | 0.00 | 3.02 | -19.87 | 3 | 0.00 |
| 18.07 | 4 | 11 | Low | 31 | 0.00 | 16 | 6.25 | 11 | 0.00 | 3.63 | -2.48 | 3 | 0.00 |
| 18.07 | 4 | 12 | Low | 32 | 3.13 | 17 | 0.00 | 10 | 0.00 | 3.95 | 4.56 | 3 | 0.00 |
| 18.07 | 5 | 13 | Low | 30 | 3.33 | 15 | -6.67 | 11 | 9.09 | 2.98 | 8.72 | 3 | 33.33 |
| 18.07 | 5 | 14 | Low | 31 | 3.23 | 16 | 6.25 | 10 | 0.00 | 3.41 | 3.52 | 2 | -50.00 |
| 18.07 | 5 | 15 | Low | 31 | 0.00 | 17 | 5.88 | 11 | 0.00 | 4.03 | 2.98 | 3 | 0.00 |
| 18.07 | 6 | 16 | Low | 31 | 3.23 | 16 | 0.00 | 10 | 0.00 | 3.62 | 3.59 | 3 | 0.00 |
| 18.07 | 6 | 17 | Low | 32 | 3.13 | 16 | 6.25 | 10 | 0.00 | 3.43 | 27.70 | 3 | 0.00 |
| 18.07 | 6 | 18 | Low | 31 | 0.00 | 16 | 0.00 | 11 | 0.00 | 4.11 | 5.11 | 3 | -33.33 |
| 18.07 | 7 | 19 | High | 30 | 0.00 | 16 | 0.00 | 13 | 7.69 | 3.83 | 3.92 | 3 | 0.00 |
| 18.07 | 7 | 20 | High | 31 | 3.23 | 17 | 0.00 | 10 | 0.00 | 3.69 | 4.34 | 3 | 0.00 |
| 18.07 | 7 | 21 | High | 30 | 0.00 | 15 | 0.00 | 11 | 0.00 | 3.74 | 5.08 | 3 | 0.00 |
| 18.07 | 8 | 22 | High | 33 | 0.00 | 18 | 5.56 | 11 | 9.09 | 4.17 | 4.80 | 4 | 0.00 |
| 18.07 | 8 | 23 | High | 30 | 0.00 | 16 | 0.00 | 10 | 0.00 | 3.69 | 4.61 | 3 | 0.00 |
| 18.07 | 8 | 24 | High | 33 | 3.03 | 16 | 0.00 | 11 | 0.00 | 4.26 | 4.69 | 3 | 0.00 |
| 18.07 | 9 | 25 | Low | 29 | 0.00 | 15 | 0.00 | 10 | 0.00 | 3.34 | 2.69 | 2 | 0.00 |
| 18.07 | 9 | 26 | Low | 33 | 3.03 | 16 | 0.00 | 10 | 0.00 | 3.69 | -0.27 | 4 | 25.00 |
| 18.07 | 9 | 27 | Low | 32 | 3.13 | 16 | 6.25 | 14 | 7.14 | 4.26 | 1.17 | 3 | -33.33 |
| 18.07 | 10 | 28 | Low | 29 | 0.00 | 15 | 0.00 | 9 | 0.00 | 2.78 | 0.72 | 2 | 0.00 |
| 18.07 | 10 | 29 | Low | 30 | 0.00 | 15 | 0.00 | 10 | 0.00 | 3.41 | 2.05 | 3 | 0.00 |
| 18.07 | 10 | 30 | Low | 30 | 3.33 | 14 | 0.00 | 11 | 0.00 | 3.29 | 13.37 | 3 | 0.00 |
| 18.07 | 11 | 31 | High | 30 | 3.33 | 16 | 0.00 | 10 | 0.00 | 3.45 | 4.35 | 3 | 33.33 |
| 18.07 | 11 | 32 | High | 29 | 0.00 | 16 | 0.00 | 10 | 0.00 | 3.35 | 3.58 | 3 | 0.00 |
| 18.07 | 11 | 33 | High | 28 | 0.00 | 15 | 0.00 | 10 | 0.00 | 3.00 | -2.00 | 2 | -50.00 |
| 18.07 | 12 | 34 | High | 30 | 0.00 | 16 | 0.00 | 11 | 0.00 | 3.69 | 2.71 | 3 | 0.00 |
| 18.07 | 12 | 35 | High | 32 | 0.00 | 16 | 0.00 | 10 | 0.00 | 3.69 | 1.36 | 3 | 0.00 |
| 18.07 | 12 | 36 | High | 29 | 0.00 | 16 | 0.00 | 11 | 0.00 | 3.61 | 1.94 | 3 | 0.00 |
| 26.07 | 1 | 1 | High | 31 | 0.00 | 18 | 0.00 | 12 | 0.00 | 4.43 | 3.16 | 3 | 0.00 |
| 26.07 | 1 | 2 | High | 32 | 0.00 | 17 | 0.00 | 10 | -10.00 | 4.02 | 2.24 | 4 | 25.00 |
| 26.07 | 1 | 3 | High | 30 | 0.00 | 17 | 0.00 | 11 | 0.00 | 4.10 | 3.17 | 3 | -33.33 |
| 26.07 | 2 | 4 | High | 30 | 3.33 | 15 | 0.00 | 10 | 0.00 | 3.50 | 22.86 | 3 | 0.00 |
| 26.07 | 2 | 5 | High | 32 | 0.00 | 17 | 5.88 | 11 | 0.00 | 4.14 | 4.35 | 4 | 25.00 |
| 26.07 | 2 | 6 | High | 31 | 0.00 | 18 | 5.56 | 11 | 0.00 | 4.56 | 4.82 | 3 | -33.33 |
| 26.07 | 3 | 7 | Low | 30 | 0.00 | 15 | -6.67 | 11 | 0.00 | 3.88 | 4.12 | 3 | 0.00 |
| 26.07 | 3 | 8 | Low | 33 | 0.00 | 16 | 0.00 | 12 | 0.00 | 4.54 | 2.86 | 4 | 0.00 |
| 26.07 | 3 | 9 | Low | 34 | 2.94 | 18 | 5.56 | 12 | 0.00 | 4.70 | 7.87 | 4 | 25.00 |
| 26.07 | 4 | 10 | Low | 32 | 0.00 | 16 | 0.00 | 10 | 0.00 | 3.78 | 20.11 | 3 | 0.00 |
| 26.07 | 4 | 11 | Low | 31 | 0.00 | 16 | 0.00 | 12 | 8.33 | 4.06 | 10.59 | 4 | 25.00 |
| 26.07 | 4 | 12 | Low | 32 | 0.00 | 17 | 0.00 | 10 | 0.00 | 4.13 | 4.36 | 3 | 0.00 |
| 26.07 | 5 | 13 | Low | 31 | 3.23 | 16 | 6.25 | 11 | 0.00 | 3.81 | 21.78 | 3 | 0.00 |
| 26.07 | 5 | 14 | Low | 31 | 0.00 | 16 | 0.00 | 10 | 0.00 | 3.77 | 9.55 | 3 | 33.33 |
| 26.07 | 5 | 15 | Low | 31 | 0.00 | 17 | 0.00 | 11 | 0.00 | 4.17 | 3.36 | 4 | 25.00 |
| 26.07 | 6 | 16 | Low | 31 | 0.00 | 16 | 0.00 | 10 | 0.00 | 3.74 | 3.21 | 3 | 0.00 |
| 26.07 | 6 | 17 | Low | 33 | 3.03 | 16 | 0.00 | 11 | 9.09 | 3.37 | -1.78 | 3 | 0.00 |
| 26.07 | 6 | 18 | Low | 32 | 3.13 | 16 | 0.00 | 12 | 8.33 | 3.73 | -10.19 | 3 | 0.00 |
| 26.07 | 7 | 19 | High | 30 | 0.00 | 16 | 0.00 | 12 | -8.33 | 3.88 | 1.29 | 3 | 0.00 |
| 26.07 | 7 | 20 | High | 31 | 0.00 | 17 | 0.00 | 10 | 0.00 | 3.81 | 3.15 | 3 | 0.00 |
| 26.07 | 7 | 21 | High | 30 | 0.00 | 15 | 0.00 | 11 | 0.00 | 3.80 | 1.58 | 4 | 25.00 |
| 26.07 | 8 | 22 | High | 34 | 2.94 | 18 | 0.00 | 11 | 0.00 | 4.37 | 4.58 | 3 | -33.33 |
| 26.07 | 8 | 23 | High | 31 | 3.23 | 17 | 5.88 | 11 | 9.09 | 3.91 | 5.63 | 3 | 0.00 |
| 26.07 | 8 | 24 | High | 33 | 0.00 | 17 | 5.88 | 11 | 0.00 | 4.46 | 4.48 | 4 | 25.00 |
| 26.07 | 9 | 25 | Low | 29 | 0.00 | 15 | 0.00 | 10 | 0.00 | 3.52 | 5.11 | 3 | 33.33 |
| 26.07 | 9 | 26 | Low | 33 | 0.00 | 16 | 0.00 | 10 | 0.00 | 3.89 | 5.14 | 3 | -33.33 |
| 26.07 | 9 | 27 | Low | 32 | 0.00 | 16 | 0.00 | 14 | 0.00 | 4.60 | 7.39 | 3 | 0.00 |
| 26.07 | 10 | 28 | Low | 29 | 0.00 | 15 | 0.00 | 9 | 0.00 | 2.79 | 0.36 | 2 | 0.00 |
| 26.07 | 10 | 29 | Low | 31 | 3.23 | 15 | 0.00 | 10 | 0.00 | 3.51 | 2.85 | 3 | 0.00 |
| 26.07 | 10 | 30 | Low | 29 | -3.45 | 14 | 0.00 | 10 | -10.00 | 3.33 | 1.20 | 3 | 0.00 |
| 26.07 | 11 | 31 | High | 31 | 3.23 | 17 | 5.88 | 10 | 0.00 | 3.78 | 8.73 | 3 | 0.00 |
| 26.07 | 11 | 32 | High | 30 | 3.33 | 16 | 0.00 | 10 | 0.00 | 3.52 | 4.83 | 2 | -50.00 |
| 26.07 | 11 | 33 | High | 28 | 0.00 | 15 | 0.00 | 10 | 0.00 | 3.08 | 2.60 | 3 | 33.33 |
| 26.07 | 12 | 34 | High | 30 | 0.00 | 16 | 0.00 | 11 | 0.00 | 3.80 | 2.89 | 3 | 0.00 |
| 26.07 | 12 | 35 | High | 32 | 0.00 | 16 | 0.00 | 10 | 0.00 | 3.83 | 3.66 | 3 | 0.00 |
| 26.07 | 12 | 36 | High | 29 | 0.00 | 16 | 0.00 | 11 | 0.00 | 3.67 | 1.63 | 3 | 0.00 |
| 16.08 | 1 | 1 | High | 32 | 3.13 | 18 | 0.00 | 12 | 0.00 | 4.79 | 7.52 | 3 | 0.00 |
| 16.08 | 1 | 2 | High | 34 | 5.88 | 19 | 10.53 | 11 | 9.09 | 4.69 | 14.29 | 4 | 0.00 |
| 16.08 | 1 | 3 | High | 31 | 3.23 | 17 | 0.00 | 12 | 8.33 | 4.46 | 8.07 | 3 | 0.00 |
| 16.08 | 2 | 4 | High | 31 | 3.23 | 16 | 6.25 | 11 | 9.09 | 3.84 | 8.85 | 3 | 0.00 |
| 16.08 | 2 | 5 | High | 33 | 3.03 | 18 | 5.56 | 11 | 0.00 | 4.60 | 10.00 | 4 | 0.00 |
| 16.08 | 2 | 6 | High | 34 | 8.82 | 19 | 5.26 | 12 | 8.33 | 5.38 | 15.24 | 4 | 25.00 |
| 16.08 | 3 | 7 | Low | 31 | 3.23 | 16 | 6.25 | 12 | 8.33 | 4.08 | 4.90 | 3 | 0.00 |
| 16.08 | 3 | 8 | Low | 33 | 0.00 | 16 | 0.00 | 12 | 0.00 | 4.64 | 2.16 | 4 | 0.00 |
| 16.08 | 3 | 9 | Low | 36 | 5.56 | 19 | 5.26 | 13 | 7.69 | 5.59 | 15.92 | 5 | 20.00 |
| 16.08 | 4 | 10 | Low | nd | nd | nd | nd | nd | nd | nd | nd | nd | nd |
| 16.08 | 4 | 11 | Low | 32 | 3.13 | 16 | 0.00 | 12 | 0.00 | 4.37 | 7.09 | 3 | -33.33 |
| 16.08 | 4 | 12 | Low | nd | nd | nd | nd | nd | nd | nd | nd | nd | nd |
| 16.08 | 5 | 13 | Low | 33 | 6.06 | 17 | 5.88 | 11 | 0.00 | 4.53 | 15.89 | 4 | 25.00 |
| 16.08 | 5 | 14 | Low | 34 | 8.82 | 17 | 5.88 | 12 | 16.67 | 4.51 | 16.41 | 4 | 25.00 |
| 16.08 | 5 | 15 | Low | 32 | 3.13 | 17 | 0.00 | 11 | 0.00 | 4.48 | 6.92 | 4 | 0.00 |
| 16.08 | 6 | 16 | Low | 32 | 3.13 | 17 | 5.88 | 10 | 0.00 | 4.00 | 6.50 | 3 | 0.00 |
| 16.08 | 6 | 17 | Low | 34 | 2.94 | 17 | 5.88 | 11 | 0.00 | 4.32 | 21.99 | 4 | 25.00 |
| 16.08 | 6 | 18 | Low | 33 | 3.03 | 17 | 5.88 | 12 | 0.00 | 4.83 | 22.77 | 4 | 25.00 |
| 16.08 | 7 | 19 | High | 30 | 0.00 | 16 | 0.00 | 12 | 0.00 | 4.01 | 3.24 | 3 | 0.00 |
| 16.08 | 7 | 20 | High | 32 | 3.13 | 17 | 0.00 | 10 | 0.00 | 4.09 | 6.85 | 3 | 0.00 |
| 16.08 | 7 | 21 | High | 31 | 3.23 | 16 | 6.25 | 11 | 0.00 | 4.04 | 5.94 | 3 | -33.33 |
| 16.08 | 8 | 22 | High | 35 | 2.86 | 18 | 0.00 | 11 | 0.00 | 4.81 | 9.15 | 4 | 25.00 |
| 16.08 | 8 | 23 | High | 33 | 6.06 | 18 | 5.56 | 11 | 0.00 | 4.54 | 13.88 | 4 | 25.00 |
| 16.08 | 8 | 24 | High | 34 | 2.94 | 17 | 0.00 | 12 | 8.33 | 4.92 | 9.35 | 4 | 0.00 |
| 16.08 | 9 | 25 | Low | 30 | 3.33 | 16 | 6.25 | 11 | 9.09 | 3.84 | 8.33 | 4 | 25.00 |
| 16.08 | 9 | 26 | Low | 34 | 2.94 | 17 | 5.88 | 11 | 9.09 | 4.22 | 7.82 | 4 | 25.00 |
| 16.08 | 9 | 27 | Low | 33 | 3.03 | 16 | 0.00 | 13 | -7.69 | 4.85 | 5.15 | 4 | 25.00 |
| 16.08 | 10 | 28 | Low | 30 | 3.33 | 15 | 0.00 | 9 | 0.00 | 3.09 | 9.71 | 3 | 33.33 |
| 16.08 | 10 | 29 | Low | 31 | 0.00 | 15 | 0.00 | 11 | 9.09 | 3.75 | 6.40 | 3 | 0.00 |
| 16.08 | 10 | 30 | Low | 30 | 3.33 | 15 | 6.67 | 11 | 9.09 | 3.56 | 6.46 | 3 | 0.00 |
| 16.08 | 11 | 31 | High | 33 | 6.06 | 18 | 5.56 | 11 | 9.09 | 4.62 | 18.18 | 3 | 0.00 |
| 16.08 | 11 | 32 | High | 32 | 6.25 | 17 | 5.88 | 11 | 9.09 | 3.21 | -9.66 | 3 | 33.33 |
| 16.08 | 11 | 33 | High | 29 | 3.45 | 15 | 0.00 | 10 | 0.00 | 3.43 | 10.20 | 2 | -50.00 |
| 16.08 | 12 | 34 | High | 31 | 3.23 | 17 | 5.88 | 11 | 0.00 | 4.08 | 6.86 | 4 | 25.00 |
| 16.08 | 12 | 35 | High | 33 | 3.03 | 16 | 0.00 | 11 | 9.09 | 4.14 | 7.49 | 3 | 0.00 |
| 16.08 | 12 | 36 | High | 29 | 0.00 | 16 | 0.00 | 11 | 0.00 | 3.83 | 4.18 | 3 | 0.00 |
| 23.08 | 1 | 1 | High | 32 | 0.00 | 18 | 0.00 | 12 | 0.00 | 4.85 | 1.24 | 4 | 25.00 |
| 23.08 | 1 | 2 | High | 35 | 2.86 | 19 | 0.00 | 12 | 8.33 | 5.00 | 6.20 | 4 | 0.00 |
| 23.08 | 1 | 3 | High | 31 | 0.00 | 17 | 0.00 | 12 | 0.00 | 4.57 | 2.41 | 4 | 25.00 |
| 23.08 | 2 | 4 | High | 31 | 0.00 | 16 | 0.00 | 11 | 0.00 | 3.93 | 2.29 | 3 | 0.00 |
| 23.08 | 2 | 5 | High | 34 | 2.94 | 17 | -5.88 | 11 | 0.00 | 4.72 | 2.54 | 4 | 0.00 |
| 23.08 | 2 | 6 | High | 34 | 0.00 | 19 | 0.00 | 12 | 0.00 | 5.57 | 3.41 | 4 | 0.00 |
| 23.08 | 3 | 7 | Low | 32 | 3.13 | 16 | 0.00 | 12 | 0.00 | 4.25 | 4.00 | 3 | 0.00 |
| 23.08 | 3 | 8 | Low | 33 | 0.00 | 16 | 0.00 | 12 | 0.00 | 4.67 | 0.64 | 3 | -33.33 |
| 23.08 | 3 | 9 | Low | 37 | 2.70 | 19 | 0.00 | 13 | 0.00 | 5.80 | 3.62 | 5 | 0.00 |
| 23.08 | 4 | 10 | Low | nd | nd | nd | nd | nd | nd | nd | nd | nd | nd |
| 23.08 | 4 | 11 | Low | 32 | 0.00 | 16 | 0.00 | 12 | 0.00 | 4.42 | 1.13 | 4 | 25.00 |
| 23.08 | 4 | 12 | Low | nd | nd | nd | nd | nd | nd | nd | nd | nd | nd |
| 23.08 | 5 | 13 | Low | 33 | 0.00 | 17 | 0.00 | 12 | 8.33 | 4.70 | 3.62 | 4 | 0.00 |
| 23.08 | 5 | 14 | Low | 34 | 0.00 | 17 | 0.00 | 12 | 0.00 | 4.64 | 2.80 | 3 | -33.33 |
| 23.08 | 5 | 15 | Low | 32 | 0.00 | 17 | 0.00 | 11 | 0.00 | 4.55 | 1.54 | 3 | -33.33 |
| 23.08 | 6 | 16 | Low | 32 | 0.00 | 17 | 0.00 | 11 | 9.09 | 4.15 | 3.61 | 3 | 0.00 |
| 23.08 | 6 | 17 | Low | 35 | 2.86 | 17 | 0.00 | 11 | 0.00 | 4.43 | 2.48 | 4 | 0.00 |
| 23.08 | 6 | 18 | Low | 33 | 0.00 | 17 | 0.00 | 12 | 0.00 | 4.87 | 0.82 | 4 | 0.00 |
| 23.08 | 7 | 19 | High | 31 | 3.23 | 16 | 0.00 | 12 | 0.00 | 4.05 | 0.99 | 3 | 0.00 |
| 23.08 | 7 | 20 | High | 32 | 0.00 | 18 | 5.56 | 11 | 9.09 | 4.20 | 2.62 | 3 | 0.00 |
| 23.08 | 7 | 21 | High | 31 | 0.00 | 16 | 0.00 | 12 | 8.33 | 4.11 | 1.70 | 3 | 0.00 |
| 23.08 | 8 | 22 | High | 35 | 0.00 | 18 | 0.00 | 11 | 0.00 | 4.95 | 2.83 | 4 | 0.00 |
| 23.08 | 8 | 23 | High | 33 | 0.00 | 18 | 0.00 | 12 | 8.33 | 4.70 | 3.40 | 4 | 0.00 |
| 23.08 | 8 | 24 | High | 34 | 0.00 | 17 | 0.00 | 12 | 0.00 | 5.07 | 2.96 | 4 | 0.00 |
| 23.08 | 9 | 25 | Low | 30 | 0.00 | 16 | 0.00 | 11 | 0.00 | 3.88 | 1.03 | 3 | -33.33 |
| 23.08 | 9 | 26 | Low | 34 | 0.00 | 17 | 0.00 | 11 | 0.00 | 3.85 | -9.61 | 3 | -33.33 |
| 23.08 | 9 | 27 | Low | 33 | 0.00 | 16 | 0.00 | 14 | 7.14 | 4.93 | 1.62 | 4 | 0.00 |
| 23.08 | 10 | 28 | Low | 30 | 0.00 | 15 | 0.00 | 9 | 0.00 | 2.71 | -14.02 | 2 | -50.00 |
| 23.08 | 10 | 29 | Low | 32 | 3.13 | 15 | 0.00 | 11 | 0.00 | 3.85 | 2.60 | 3 | 0.00 |
| 23.08 | 10 | 30 | Low | 30 | 0.00 | 15 | 0.00 | 11 | 0.00 | 3.55 | -0.28 | 3 | 0.00 |
| 23.08 | 11 | 31 | High | 33 | 0.00 | 18 | 0.00 | 11 | 0.00 | 4.79 | 3.55 | 4 | 25.00 |
| 23.08 | 11 | 32 | High | 32 | 0.00 | 18 | 5.56 | 11 | 0.00 | 4.46 | 28.03 | 4 | 25.00 |
| 23.08 | 11 | 33 | High | 29 | 0.00 | 15 | 0.00 | 10 | 0.00 | 3.49 | 1.72 | 3 | 33.33 |
| 23.08 | 12 | 34 | High | 32 | 3.13 | 17 | 0.00 | 11 | 0.00 | 4.19 | 2.63 | 3 | -33.33 |
| 23.08 | 12 | 35 | High | 33 | 0.00 | 16 | 0.00 | 11 | 0.00 | 4.23 | 2.13 | 3 | 0.00 |
| 23.08 | 12 | 36 | High | 30 | 3.33 | 16 | 0.00 | 11 | 0.00 | 3.90 | 1.79 | 3 | 0.00 |
| 30.08 | 1 | 1 | High | 33 | 3.03 | 18 | 0.00 | 12 | 0.00 | 4.96 | 2.22 | 4 | 0.00 |
| 30.08 | 1 | 2 | High | 36 | 2.78 | 18 | -5.56 | 12 | 0.00 | 4.81 | -3.95 | 5 | 20.00 |
| 30.08 | 1 | 3 | High | 32 | 3.13 | 18 | 5.56 | 12 | 0.00 | 4.70 | 2.77 | 4 | 0.00 |
| 30.08 | 2 | 4 | High | 32 | 3.13 | 17 | 5.88 | 11 | 0.00 | 4.11 | 4.38 | 3 | 0.00 |
| 30.08 | 2 | 5 | High | 34 | 0.00 | 18 | 5.56 | 12 | 8.33 | 4.52 | -4.42 | 3 | -33.33 |
| 30.08 | 2 | 6 | High | 35 | 2.86 | 20 | 5.00 | 13 | 7.69 | 5.80 | 3.97 | 4 | 0.00 |
| 30.08 | 3 | 7 | Low | 32 | 0.00 | 16 | 0.00 | 12 | 0.00 | 4.47 | 4.92 | 3 | 0.00 |
| 30.08 | 3 | 8 | Low | 33 | 0.00 | 16 | 0.00 | 12 | 0.00 | 4.16 | -12.26 | 4 | 25.00 |
| 30.08 | 3 | 9 | Low | 37 | 0.00 | 19 | 0.00 | 13 | 0.00 | 6.07 | 4.45 | 5 | 0.00 |
| 30.08 | 4 | 10 | Low | nd | nd | nd | nd | nd | nd | nd | nd | nd | nd |
| 30.08 | 4 | 11 | Low | 33 | 3.03 | 17 | 5.88 | 12 | 0.00 | 4.63 | 4.54 | 4 | 0.00 |
| 30.08 | 4 | 12 | Low | nd | nd | nd | nd | nd | nd | nd | nd | nd | nd |
| 30.08 | 5 | 13 | Low | 34 | 2.94 | 17 | 0.00 | 12 | 0.00 | 4.78 | 1.67 | 4 | 0.00 |
| 30.08 | 5 | 14 | Low | 34 | 0.00 | 18 | 5.56 | 12 | 0.00 | 4.83 | 3.93 | 4 | 25.00 |
| 30.08 | 5 | 15 | Low | 33 | 3.03 | 17 | 0.00 | 11 | 0.00 | 4.62 | 1.52 | 4 | 25.00 |
| 30.08 | 6 | 16 | Low | nd | nd | nd | nd | nd | nd | nd | nd | nd | nd |
| 30.08 | 6 | 17 | Low | nd | nd | nd | nd | nd | nd | nd | nd | nd | nd |
| 30.08 | 6 | 18 | Low | nd | nd | nd | nd | nd | nd | nd | nd | nd | nd |
| 30.08 | 7 | 19 | High | 31 | 0.00 | 16 | 0.00 | 12 | 0.00 | 4.08 | 0.74 | 3 | 0.00 |
| 30.08 | 7 | 20 | High | 33 | 3.03 | 18 | 0.00 | 11 | 0.00 | 4.30 | 2.33 | 3 | 0.00 |
| 30.08 | 7 | 21 | High | 31 | 0.00 | 16 | 0.00 | 12 | 0.00 | 4.24 | 3.07 | 3 | 0.00 |
| 30.08 | 8 | 22 | High | 35 | 0.00 | 18 | 0.00 | 11 | 0.00 | 5.07 | 2.37 | 4 | 0.00 |
| 30.08 | 8 | 23 | High | 34 | 2.94 | 18 | 0.00 | 12 | 0.00 | 4.90 | 4.08 | 4 | 0.00 |
| 30.08 | 8 | 24 | High | 35 | 2.86 | 18 | 5.56 | 12 | 0.00 | 5.17 | 1.93 | 5 | 20.00 |
| 30.08 | 9 | 25 | Low | 31 | 3.23 | 17 | 5.88 | 11 | 0.00 | 3.98 | 2.51 | 3 | 0.00 |
| 30.08 | 9 | 26 | Low | 35 | 2.86 | 16 | -6.25 | 11 | 0.00 | 4.77 | 19.29 | 4 | 25.00 |
| 30.08 | 9 | 27 | Low | 33 | 0.00 | 16 | 0.00 | 14 | 0.00 | 5.01 | 1.60 | 4 | 0.00 |
| 30.08 | 10 | 28 | Low | 30 | 0.00 | 15 | 0.00 | 9 | 0.00 | 3.24 | 16.36 | 2 | 0.00 |
| 30.08 | 10 | 29 | Low | 32 | 0.00 | 16 | 6.25 | 11 | 0.00 | 3.98 | 3.27 | 3 | 0.00 |
| 30.08 | 10 | 30 | Low | 31 | 3.23 | 15 | 0.00 | 11 | 0.00 | 3.59 | 1.11 | 3 | 0.00 |
| 30.08 | 11 | 31 | High | 34 | 2.94 | 19 | 5.26 | 11 | 0.00 | 5.01 | 4.39 | 4 | 0.00 |
| 30.08 | 11 | 32 | High | 33 | 3.03 | 18 | 0.00 | 11 | 0.00 | 4.73 | 5.71 | 4 | 0.00 |
| 30.08 | 11 | 33 | High | 30 | 3.33 | 15 | 0.00 | 10 | 0.00 | 3.60 | 3.06 | 3 | 0.00 |
| 30.08 | 12 | 34 | High | 32 | 0.00 | 17 | 0.00 | 12 | 8.33 | 4.33 | 3.23 | 4 | 25.00 |
| 30.08 | 12 | 35 | High | 34 | 2.94 | 17 | 5.88 | 11 | 0.00 | 4.36 | 2.98 | 4 | 25.00 |
| 30.08 | 12 | 36 | High | 30 | 0.00 | 16 | 0.00 | 11 | 0.00 | 4.02 | 2.99 | 3 | 0.00 |
| 06.09 | 1 | 1 | High | 33 | 0.00 | 19 | 5.26 | 12 | 0.00 | 5.09 | 2.55 | 4 | 0.00 |
| 06.09 | 1 | 2 | High | 36 | 0.00 | 20 | 10.00 | 12 | 0.00 | 5.49 | 12.39 | 5 | 0.00 |
| 06.09 | 1 | 3 | High | 32 | 0.00 | 18 | 0.00 | 12 | 0.00 | 4.80 | 2.08 | 4 | 0.00 |
| 06.09 | 2 | 4 | High | 32 | 0.00 | 17 | 0.00 | 12 | 8.33 | 4.31 | 4.64 | 4 | 25.00 |
| 06.09 | 2 | 5 | High | 35 | 2.86 | 18 | 0.00 | 12 | 0.00 | 5.06 | 10.67 | 4 | 25.00 |
| 06.09 | 2 | 6 | High | 35 | 0.00 | 20 | 0.00 | 13 | 0.00 | 6.03 | 3.81 | 5 | 20.00 |
| 06.09 | 3 | 7 | Low | 33 | 3.03 | 17 | 5.88 | 13 | 7.69 | 4.87 | 8.21 | 4 | 25.00 |
| 06.09 | 3 | 8 | Low | 33 | 0.00 | 17 | 5.88 | 13 | 7.69 | 4.89 | 14.93 | 4 | 0.00 |
| 06.09 | 3 | 9 | Low | 39 | 5.13 | 20 | 5.00 | 14 | 7.14 | 6.63 | 8.45 | 5 | 0.00 |
| 06.09 | 4 | 10 | Low | nd | nd | nd | nd | nd | nd | nd | nd | nd | nd |
| 06.09 | 4 | 11 | Low | 33 | 0.00 | 17 | 0.00 | 13 | 7.69 | 4.85 | 4.54 | 4 | 0.00 |
| 06.09 | 4 | 12 | Low | nd | nd | nd | nd | nd | nd | nd | nd | nd | nd |
| 06.09 | 5 | 13 | Low | 34 | 0.00 | 18 | 5.56 | 12 | 0.00 | 5.04 | 5.16 | 4 | 0.00 |
| 06.09 | 5 | 14 | Low | 35 | 2.86 | 18 | 0.00 | 12 | 0.00 | 5.07 | 4.73 | 4 | 0.00 |
| 06.09 | 5 | 15 | Low | 33 | 0.00 | 17 | 0.00 | 11 | 0.00 | 4.74 | 2.53 | 4 | 0.00 |
| 06.09 | 6 | 16 | Low | nd | nd | nd | nd | nd | nd | nd | nd | nd | nd |
| 06.09 | 6 | 17 | Low | nd | nd | nd | nd | nd | nd | nd | nd | nd | nd |
| 06.09 | 6 | 18 | Low | nd | nd | nd | nd | nd | nd | nd | nd | nd | nd |
| 06.09 | 7 | 19 | High | 31 | 0.00 | 16 | 0.00 | 12 | 0.00 | 4.18 | 2.39 | 3 | 0.00 |
| 06.09 | 7 | 20 | High | 33 | 0.00 | 18 | 0.00 | 11 | 0.00 | 4.49 | 4.23 | 4 | 25.00 |
| 06.09 | 7 | 21 | High | 31 | 0.00 | 16 | 0.00 | 12 | 0.00 | 4.31 | 1.62 | 4 | 25.00 |
| 06.09 | 8 | 22 | High | 36 | 2.78 | 19 | 5.26 | 12 | 8.33 | 5.30 | 4.34 | 4 | 0.00 |
| 06.09 | 8 | 23 | High | 34 | 0.00 | 18 | 0.00 | 12 | 0.00 | 5.09 | 3.73 | 4 | 0.00 |
| 06.09 | 8 | 24 | High | 35 | 0.00 | 18 | 0.00 | 12 | 0.00 | 5.22 | 0.96 | 4 | -25.00 |
| 06.09 | 9 | 25 | Low | 31 | 0.00 | 17 | 0.00 | 11 | 0.00 | 4.12 | 3.40 | 4 | 25.00 |
| 06.09 | 9 | 26 | Low | 36 | 2.78 | 18 | 11.11 | 11 | 0.00 | 5.02 | 4.98 | 5 | 20.00 |
| 06.09 | 9 | 27 | Low | 33 | 0.00 | 16 | 0.00 | 14 | 0.00 | 5.13 | 2.34 | 4 | 0.00 |
| 06.09 | 10 | 28 | Low | 31 | 3.23 | 15 | 0.00 | 9 | 0.00 | 3.37 | 3.86 | 3 | 33.33 |
| 06.09 | 10 | 29 | Low | 32 | 0.00 | 16 | 0.00 | 11 | 0.00 | 4.13 | 3.63 | 4 | 25.00 |
| 06.09 | 10 | 30 | Low | 31 | 0.00 | 15 | 0.00 | 11 | 0.00 | 3.77 | 4.77 | 3 | 0.00 |
| 06.09 | 11 | 31 | High | 35 | 2.86 | 19 | 0.00 | 12 | 8.33 | 5.32 | 5.83 | 4 | 0.00 |
| 06.09 | 11 | 32 | High | 34 | 2.94 | 19 | 5.26 | 11 | 0.00 | 5.08 | 6.89 | 4 | 0.00 |
| 06.09 | 11 | 33 | High | 30 | 0.00 | 15 | 0.00 | 10 | 0.00 | 3.70 | 2.70 | 3 | 0.00 |
| 06.09 | 12 | 34 | High | 32 | 0.00 | 17 | 0.00 | 12 | 0.00 | 4.49 | 3.56 | 3 | -33.33 |
| 06.09 | 12 | 35 | High | 34 | 0.00 | 17 | 0.00 | 11 | 0.00 | 4.46 | 2.24 | 3 | -33.33 |
| 06.09 | 12 | 36 | High | 30 | 0.00 | 17 | 5.88 | 11 | 0.00 | 4.15 | 3.13 | 3 | 0.00 |
| 13.09 | 1 | 1 | High | 33 | 0.00 | 19 | 0.00 | 13 | 7.69 | 5.19 | 1.93 | 4 | 0.00 |
| 13.09 | 1 | 2 | High | 37 | 2.70 | 20 | 0.00 | 12 | 0.00 | 5.84 | 5.99 | 5 | 0.00 |
| 13.09 | 1 | 3 | High | 32 | 0.00 | 17 | -5.88 | 12 | 0.00 | 4.92 | 2.44 | 4 | 0.00 |
| 13.09 | 2 | 4 | High | 33 | 3.03 | 17 | 0.00 | 12 | 0.00 | 4.54 | 5.07 | 4 | 0.00 |
| 13.09 | 2 | 5 | High | 35 | 0.00 | 18 | 0.00 | 12 | 0.00 | 5.17 | 2.13 | 5 | 20.00 |
| 13.09 | 2 | 6 | High | 36 | 2.78 | 20 | 0.00 | 13 | 0.00 | 6.30 | 4.29 | 5 | 0.00 |
| 13.09 | 3 | 7 | Low | 34 | 2.94 | 18 | 5.56 | 13 | 0.00 | 4.98 | 2.21 | 4 | 0.00 |
| 13.09 | 3 | 8 | Low | 34 | 2.94 | 17 | 0.00 | 13 | 0.00 | 5.04 | 2.98 | 4 | 0.00 |
| 13.09 | 3 | 9 | Low | 40 | 2.50 | 21 | 4.76 | 14 | 0.00 | 7.20 | 7.92 | 6 | 16.67 |
| 13.09 | 4 | 10 | Low | nd | nd | nd | nd | nd | nd | nd | nd | nd | nd |
| 13.09 | 4 | 11 | Low | 34 | 2.94 | 17 | 0.00 | 13 | 0.00 | 5.06 | 4.15 | 4 | 0.00 |
| 13.09 | 4 | 12 | Low | nd | nd | nd | nd | nd | nd | nd | nd | nd | nd |
| 13.09 | 5 | 13 | Low | 35 | 2.86 | 18 | 0.00 | 12 | 0.00 | 5.29 | 4.73 | 4 | 0.00 |
| 13.09 | 5 | 14 | Low | 35 | 0.00 | 18 | 0.00 | 12 | 0.00 | 5.28 | 3.98 | 4 | 0.00 |
| 13.09 | 5 | 15 | Low | 33 | 0.00 | 17 | 0.00 | 11 | 0.00 | 4.86 | 2.47 | 4 | 0.00 |
| 13.09 | 6 | 16 | Low | nd | nd | nd | nd | nd | nd | nd | nd | nd | nd |
| 13.09 | 6 | 17 | Low | nd | nd | nd | nd | nd | nd | nd | nd | nd | nd |
| 13.09 | 6 | 18 | Low | nd | nd | nd | nd | nd | nd | nd | nd | nd | nd |
| 13.09 | 7 | 19 | High | 31 | 0.00 | 16 | 0.00 | 12 | 0.00 | 4.23 | 1.18 | 4 | 25.00 |
| 13.09 | 7 | 20 | High | 33 | 0.00 | 18 | 0.00 | 11 | 0.00 | 4.56 | 1.54 | 4 | 0.00 |
| 13.09 | 7 | 21 | High | 31 | 0.00 | 16 | 0.00 | 11 | -9.09 | 4.39 | 1.82 | 4 | 0.00 |
| 13.09 | 8 | 22 | High | 36 | 0.00 | 19 | 0.00 | 12 | 0.00 | 5.49 | 3.46 | 4 | 0.00 |
| 13.09 | 8 | 23 | High | 35 | 2.86 | 19 | 5.26 | 12 | 0.00 | 5.30 | 3.96 | 5 | 20.00 |
| 13.09 | 8 | 24 | High | 36 | 2.78 | 18 | 0.00 | 12 | 0.00 | 5.49 | 4.92 | 4 | 0.00 |
| 13.09 | 9 | 25 | Low | 32 | 3.13 | 17 | 0.00 | 11 | 0.00 | 4.29 | 3.96 | 3 | -33.33 |
| 13.09 | 9 | 26 | Low | 36 | 0.00 | 18 | 0.00 | 12 | 8.33 | 5.09 | 1.38 | 4 | -25.00 |
| 13.09 | 9 | 27 | Low | 34 | 2.94 | 17 | 5.88 | 14 | 0.00 | 5.34 | 3.93 | 4 | 0.00 |
| 13.09 | 10 | 28 | Low | 31 | 0.00 | 16 | 6.25 | 10 | 10.00 | 3.53 | 4.53 | 3 | 0.00 |
| 13.09 | 10 | 29 | Low | 33 | 3.03 | 17 | 5.88 | 11 | 0.00 | 4.34 | 4.84 | 3 | -33.33 |
| 13.09 | 10 | 30 | Low | 31 | 0.00 | 15 | 0.00 | 11 | 0.00 | 3.90 | 3.33 | 3 | 0.00 |
| 13.09 | 11 | 31 | High | 35 | 0.00 | 19 | 0.00 | 12 | 0.00 | 5.55 | 4.14 | 4 | 0.00 |
| 13.09 | 11 | 32 | High | 35 | 2.86 | 19 | 0.00 | 12 | 8.33 | 5.40 | 5.93 | 5 | 20.00 |
| 13.09 | 11 | 33 | High | 30 | 0.00 | 16 | 6.25 | 11 | 9.09 | 3.84 | 3.65 | 3 | 0.00 |
| 13.09 | 12 | 34 | High | 32 | 0.00 | 18 | 5.56 | 12 | 0.00 | 4.65 | 3.44 | 4 | 25.00 |
| 13.09 | 12 | 35 | High | 34 | 0.00 | 17 | 0.00 | 11 | 0.00 | 4.53 | 1.55 | 4 | 25.00 |
| 13.09 | 12 | 36 | High | 30 | 0.00 | 17 | 0.00 | 11 | 0.00 | 4.33 | 4.16 | 3 | 0.00 |
| 20.09 | 1 | 1 | High | 33 | 0.00 | 19 | 0.00 | 13 | 0.00 | 5.32 | 2.44 | 4 | 0.00 |
| 20.09 | 1 | 2 | High | 37 | 0.00 | 20 | 0.00 | 12 | 0.00 | 5.87 | 0.51 | 5 | 0.00 |
| 20.09 | 1 | 3 | High | 32 | 0.00 | 18 | 5.56 | 12 | 0.00 | 5.00 | 1.60 | 4 | 0.00 |
| 20.09 | 2 | 4 | High | 33 | 0.00 | 17 | 0.00 | 12 | 0.00 | 4.76 | 4.62 | 4 | 0.00 |
| 20.09 | 2 | 5 | High | 35 | 0.00 | 18 | 0.00 | 12 | 0.00 | 5.31 | 2.64 | 4 | -25.00 |
| 20.09 | 2 | 6 | High | 36 | 0.00 | 20 | 0.00 | 13 | 0.00 | 6.42 | 1.87 | 5 | 0.00 |
| 20.09 | 3 | 7 | Low | 34 | 0.00 | 18 | 0.00 | 13 | 0.00 | 5.17 | 3.68 | 4 | 0.00 |
| 20.09 | 3 | 8 | Low | 34 | 0.00 | 17 | 0.00 | 13 | 0.00 | 5.23 | 3.63 | 4 | 0.00 |
| 20.09 | 3 | 9 | Low | 40 | 0.00 | 21 | 0.00 | 14 | 0.00 | 7.54 | 4.51 | 7 | 14.29 |
| 20.09 | 4 | 10 | Low | nd | nd | nd | nd | nd | nd | nd | nd | nd | nd |
| 20.09 | 4 | 11 | Low | 34 | 0.00 | 17 | 0.00 | 13 | 0.00 | 5.33 | 5.07 | 5 | 20.00 |
| 20.09 | 4 | 12 | Low | nd | nd | nd | nd | nd | nd | nd | nd | nd | nd |
| 20.09 | 5 | 13 | Low | 35 | 0.00 | 18 | 0.00 | 12 | 0.00 | 5.48 | 3.47 | 5 | 20.00 |
| 20.09 | 5 | 14 | Low | 36 | 2.78 | 18 | 0.00 | 13 | 7.69 | 5.48 | 3.65 | 5 | 20.00 |
| 20.09 | 5 | 15 | Low | 33 | 0.00 | 17 | 0.00 | 11 | 0.00 | 4.99 | 2.61 | 4 | 0.00 |
| 20.09 | 6 | 16 | Low | nd | nd | nd | nd | nd | nd | nd | nd | nd | nd |
| 20.09 | 6 | 17 | Low | nd | nd | nd | nd | nd | nd | nd | nd | nd | nd |
| 20.09 | 6 | 18 | Low | nd | nd | nd | nd | nd | nd | nd | nd | nd | nd |
| 20.09 | 7 | 19 | High | 31 | 0.00 | 17 | 5.88 | 12 | 0.00 | 4.33 | 2.31 | 3 | -33.33 |
| 20.09 | 7 | 20 | High | 33 | 0.00 | 18 | 0.00 | 11 | 0.00 | 4.75 | 4.00 | 4 | 0.00 |
| 20.09 | 7 | 21 | High | 32 | 3.13 | 16 | 0.00 | 12 | 8.33 | 4.44 | 1.13 | 3 | -33.33 |
| 20.09 | 8 | 22 | High | 37 | 2.70 | 19 | 0.00 | 12 | 0.00 | 5.66 | 3.00 | 5 | 20.00 |
| 20.09 | 8 | 23 | High | 35 | 0.00 | 18 | -5.56 | 12 | 0.00 | 5.46 | 2.93 | 4 | -25.00 |
| 20.09 | 8 | 24 | High | 36 | 0.00 | 18 | 0.00 | 12 | 0.00 | 5.60 | 1.96 | 4 | 0.00 |
| 20.09 | 9 | 25 | Low | 32 | 0.00 | 17 | 0.00 | 11 | 0.00 | 4.42 | 2.94 | 4 | 25.00 |
| 20.09 | 9 | 26 | Low | 36 | 0.00 | 18 | 0.00 | 11 | -9.09 | 5.31 | 4.14 | 4 | 0.00 |
| 20.09 | 9 | 27 | Low | 34 | 0.00 | 17 | 0.00 | 14 | 0.00 | 5.49 | 2.73 | 5 | 20.00 |
| 20.09 | 10 | 28 | Low | 31 | 0.00 | 16 | 0.00 | 9 | -11.11 | 3.65 | 3.29 | 3 | 0.00 |
| 20.09 | 10 | 29 | Low | 33 | 0.00 | 16 | -6.25 | 11 | 0.00 | 4.17 | -4.08 | 4 | 25.00 |
| 20.09 | 10 | 30 | Low | 31 | 0.00 | 15 | 0.00 | 11 | 0.00 | 3.66 | -6.56 | 3 | 0.00 |
| 20.09 | 11 | 31 | High | 35 | 0.00 | 19 | 0.00 | 12 | 0.00 | 5.70 | 2.63 | 5 | 20.00 |
| 20.09 | 11 | 32 | High | 35 | 0.00 | 19 | 0.00 | 12 | 0.00 | 5.59 | 3.40 | 4 | -25.00 |
| 20.09 | 11 | 33 | High | 31 | 3.23 | 16 | 0.00 | 11 | 0.00 | 3.93 | 2.29 | 3 | 0.00 |
| 20.09 | 12 | 34 | High | 33 | 3.03 | 18 | 0.00 | 12 | 0.00 | 4.75 | 2.11 | 4 | 0.00 |
| 20.09 | 12 | 35 | High | 34 | 0.00 | 17 | 0.00 | 11 | 0.00 | 4.61 | 1.74 | 4 | 0.00 |
| 20.09 | 12 | 36 | High | 31 | 3.23 | 17 | 0.00 | 11 | 0.00 | 4.42 | 2.04 | 3 | 0.00 |
