# Supplementary material for: Low to moderate wave exposure did not impact blue mussel (Mytilus edulis) growth in a mesocosm study
Source: PLoS One. 2024 Dec 5;19(12):e0315136. doi: 10.1371/journal.pone.0315136 (PMC11620627; doi:10.1371/journal.pone.0315136)
Supplement: Attachment — Submitted filename: Response to Reviewers.pdf [file pone.0315136.s003.pdf]

Our response to the reviewers

Dear reviewers,

Thanks for your reply to our manuscript. Both reviewers still recommend that we create a figure, and some text to follow the figure, on the change in the mussel measurements through time comparing the high wave treatment with the low wave treatment. We agree on this, and we have included a figure on changes is weight over time. We started by making a correlation matrix and found weight to be the parameter that correlated the most with the others. We believe that it is enough to include one figure, and weight was therefore selected. If you want us to include a similar figure on all parameters, we will do that. The figure shows the difference between the high and the low wave basins.

I am sorry for missing to write the repository name and DOI number of the dataset in the Data Availability Statement. The DOI has been included in the reference list in the manuscript and is now also stated in the Data Availability Statement.

Regards, Trine
