# Supplementary material for: Low to moderate wave exposure did not impact blue mussel (Mytilus edulis) growth in a mesocosm study
Source: PLoS One. 2024 Dec 5;19(12):e0315136. doi: 10.1371/journal.pone.0315136 (PMC11620627; doi:10.1371/journal.pone.0315136)
Supplement: Attachment — Submitted filename: Response to Reviewers.docx [file pone.0315136.s002.docx]

**Our response to comments regarding Journal Requirements:**

**1. PLOS ONE's requirements**

When submitting your revision, we need you to address these additional requirements.

Please ensure that your manuscript meets PLOS ONE's style requirements, including those for file naming. The PLOS ONE style templates can be found at

https://journals.plos.org/plosone/s/file?id=wjVg/PLOSOne_formatting_sample_main_body.pdf and

https://journals.plos.org/plosone/s/file?id=ba62/PLOSOne_formatting_sample_title_authors_affiliations.pdf

- **Our response**: We have changed the heading font size, the format of the figure title, the figure and the tables. We have deleted information about the funding in the Acknowledgement. The citations in the text have been changed so that they are numbers in the text, as listed in the style requirements. I turned off “track changes” when changing the order in the reference list, as the reference list changed totally all together as a result of this change.

**2. Permits**

In your Methods section, please provide additional information regarding the permits you obtained for the work. Please ensure you have included the full name of the authority that approved the field site access and, if no permits were required, a brief statement explaining why.

- **Our response**: In the Method chapter, we have now added a text in the method about the necessary approvals and the reason for the lack of need for an approval from the research ethics committees

**3. Thank you for stating the following financial disclosure:**

The project (COASTFRAG) was funded (2021-ongoing) by the Research Council of Norway, grant number 342628/L10 (project number 314314), and the EU grant AQUACOSM-plus (TA-project MESOFRAG, H2020-INFRAIA-Project No 871081). Additional funding was provided by the Norwegian Institute for Water Research. Please state what role the funders took in the study. If the funders had no role, please state: "The funders had no role in study design, data collection and analysis, decision to publish, or preparation of the manuscript.". If this statement is not correct you must amend it as needed.

- **Our response**: Yes, the text should read like this: The project (COASTFRAG) was funded (2021-ongoing) by the Research Council of Norway, grant number 342628/L10 (project number 314314), and the EU grant AQUACOSM-plus (TA-project MESOFRAG, H2020-INFRAIA-Project No 871081). Additional funding was provided by the Norwegian Institute for Water Research. The funders had no role in study design, data collection and analysis, decision to publish, or preparation of the manuscript.

Please include this amended Role of Funder statement in your cover letter; we will change the online submission form on your behalf.

- **Our response**: This has been included in the cover letter.

**4. Thank you for stating the following in the Acknowledgments Section of your manuscript:**

Thanks to the Research Council of Norway, the EU project AQUACOSM-plus and NIVA for funding. We are grateful to Benjamin Kupilaas (NIVA) for all the help getting the AQUACOSM-plus grant. The study took place at the NIVA Solbergstrand Experimental Facility, and we want to thank all the staff at Solbergstrand for their help and support. We note that you have provided funding information that is not currently declared in your Funding Statement. However, funding information should not appear in the Acknowledgments section or other areas of your manuscript. We will only publish funding information present in the Funding Statement section of the online submission form.

- **Our response**: We have now included all the funders in the Funding Statement section of the online submission form

Please remove any funding-related text from the manuscript and let us know how you would like to update your Funding Statement. Currently, your Funding Statement reads as follows:

The project (COASTFRAG) was funded (2021-ongoing) by the Research Council of Norway, grant number 342628/L10 (project number 314314), and the EU grant AQUACOSM-plus (TA-project MESOFRAG, H2020-INFRAIA-Project No 871081). Additional funding was provided by the Norwegian Institute for Water Research.

- **Our response**: I have included the text in the cover letter.

Please include your amended statements within your cover letter; we will change the online submission form on your behalf.

- **Our response**: Yes, this has now been included in the cover letter

**5. Data to be made publicly available.**

When completing the data availability statement of the submission form, you indicated that you will make your data available on acceptance. We strongly recommend all authors decide on a data sharing plan before acceptance, as the process can be lengthy and hold up publication timelines. Please note that, though access restrictions are acceptable now, your entire data will need to be made freely accessible if your manuscript is accepted for publication. This policy applies to all data except where public deposition would breach compliance with the protocol approved by your research ethics board. If you are unable to adhere to our open data policy, please kindly revise your statement to explain your reasoning and we will seek the editor's input on an exemption. Please be assured that, once you have provided your new statement, the assessment of your exemption will not hold up the peer review process.

- **Our response**: I am so sorry for not sending the data over. The plan (that I thought we followed) was to send the dataset over for the reviewers to see. But somehow, we made a mistake here, and the data was not submitted along with the manuscript. I apologize for this, particularly to the reviewers, as this has made their job much more difficult. I have now submitted the data (Lukić et al. 2024a) to Zenodo, and the doi is included in the reference list. We have also included the data as Supporting Information in a table (“S1 Table”).

**Reviewer #1 comments**

**General Comments:**
The paper is very concise and the results were not the expected according to the authors, however the experiment was run properly, despite the paper can be improved. I recommend major revisions.

- **Our response**: Thanks for all the useful comments, and I have answered them one by one below.

**Specific Comments:**

2) In the materials and methods is not well described the experimental set-up. In the abstract the authors mention that from the 12 mesocosms, 6 were for the “high wave exposure” while the other 6 were for the “low wave exposure”, but in the M&M this is not mentioned. Also, in this section is not described the size or age of the mussels used for the experiment. This should be described also.
   - **Our response**: I made the description of the wave levels in the basins clearer; I hope the information about the basins and the waves are easier to understand now. We have also described how the blue mussels entered the basins
3) In the results section, surprisingly, are not shown any results, including graphs with the evolution of mussel measurements for the different treatments. It is just represented the statistical results. But in my opinion, it is missing some graphical representation of the results, that can be complemented with the stats.
   - **Our response**: And for this I must apologize. I am so sorry for not sending the data over. The plan (that I thought we followed) was to send the dataset over for the reviewers to see. But somehow, we made a mistake here, and the data was not submitted along with the manuscript. I apologize for this, as this has made your job much more difficult. I have now submitted the data (Lukić et al. 2024a) to Zenodo, and the doi is included in the reference list. The data can therefore now be accessed. We have also included the data as Supporting Information in a table (“S1 Table”). As the data only show that the blue mussels get bigger with time (we did not find any effect of wave level), we have not included any figures. There were no significant results linking wave exposure to any of the measured parameters, so we did not want to include many figures that did not show anything. I hope you agree.
4) In the stats tables, I do not understand the advantage of studying the percentage change of the different parameters from one time to the next over the 13 weeks (table 1) and from the start to the end of the study (table 2). This should be better explained.
   - **Our response**: The last part first. I have now removed the table (Table 2) with the results from the analyses of the change in the parameters from the beginning to the end of the 13 weeks. I agree, it does not give us any more information that the data in Table 1 provides. In Table 1 have still kept the results for the % change. The reason for this is that we thought that maybe a rate of change (measured as the percentage) would provide us with some results that analysing the absolute value did not. As it was, we did not get any other results, but we still hope that we can keep this in the table.
5) The discussion is quite superficial. The authors could improve it with other case studies to compare with.
   - **Our response**: Reviewer 2 also had a similar point and wanted us to go more in depth into the two hypotheses for the negative results presented. We have therefore changed the discussion to include this (hypothesis 1: it is actually no difference when effects of other relevant variables are removed, hypothesis 2: the waves used in the study were not strong enough to generate a measurable effect).
6) Figure1 legend appears in the middle of the text, apart from the figure. Please check.
   - **Our response**: The figure text is placed just after where it the figure is mentioned in the text. This is where the figure will be placed, but now the figure in not placed in the manuscript, as this is how we have understood that is the correct way when submitting the manuscript. If the Figure is to be placed in the text when submitting the manuscript, we will of course to this.
7) In the discussion, line 130, please delete the word “these”. It should be read “influence on other processes” in spite of “influence on these other processes”.
   - **Our response**: Yes, that is correct, we have deleted “these”
8) Line 135, the sentence is confusing. Please rephrase.
   - **Our response**: I have rewritten this part of the text, hopefully making the discussion about the lack of effect dues to the small difference in wave levels I bit clearer.

**Reviewer #2 comments**

The experimental design and statistical methods appear to be sound, but I have two major concerns about the manuscript:

My first concern is the absence of results in the manuscript. I am not referring to the lack of significant differences; publishing negative results is valuable. The problem is that none of the data from the experiments appear in the paper, either in table or plot form. Statistical tests are presented, but these are essentially meaningless without seeing the values on which they are based. The manuscript cites a link to a Zenodo database, but these data do not seem to be available to reviewers, and they should be included within the text of the manuscript rather than via an external link, preferably in the form of plots. The authors write that they "did not aim to study the growth patterns of blue mussels", but this is what was measured, and the results are needed to understand the conclusions of the manuscript and could also be useful in other contexts.

- **Our response**: And for this I must apologize. I am so sorry for not sending the data over. The plan (that I thought we followed) was to send the dataset over for the reviewers to see. But somehow, we made a mistake here, and the data was not submitted along with the manuscript. I apologize for this, as this has made your job much more difficult. I have now submitted the data (Lukić et al. 2024) to Zenodo, and the doi is included in the reference list. The data can therefore now be accessed. We have also included the data as Supporting Information in a table (“S1 Table”).

My second concern is with the interpretation of the results. There are two alternative hypotheses for the negative results presented. The first is that there really is no effect of wave exposure on mussel growth when the effects of other relevant variables, including predation and food availability, are removed. The second is that the waves used in the study were not strong enough to generate a measurable effect. These two alternatives should be explicitly explored in the discussion and conclusion section and included in the abstract. There is likely information in the literature supporting one hypothesis over the other. For example: How did the strength of your wave exposures compare to those from previous studies? How did phytoplankton concentrations compare? It is worth exploring this further because if there really is no effect of waves on the growth absent the other factors, this could be an interesting finding. Overall, more effort should be devoted to putting the results in the context of previous work.

- **Our response**: Reviewer 1 has a similar point, stating that the discussion is quite superficial and that we should improve it with other case studies to compare with. We have therefore changed the discussion quite a lot. We have discussed the two theories for why we do not find any effects of wave exposure, including going in more detail into the discussion of other findings and the co-varying factors that other studies often have (with moth sedimentation, predation and competition varying with wave exposure. I hope that our discussion is better now.

I believe that addressing these two concerns could produce a much stronger manuscript. While no significant differences were found between treatments, this can still be an interesting and useful result if presented in the right way.

- **Our response**: We hope that we have responded to these to concerns in a way that you find sufficient. We believe that the manuscript now reads much better, and we hope that you agree.

Minor comments:

- Line 21: ..."a" simple, but controlled, mesocosm study...
  - **Our response**: Corrected
- Line 45: Should this be "remove sediment" instead of "remove sedimentation"?
  - **Our response**: Yes, when have changed in to “remove sediments”
- Line 129: ...impact of "wave" exposure...
  - **Our response**: Corrected
