# Supplementary material for: Low to moderate wave exposure did not impact blue mussel (Mytilus edulis) growth in a mesocosm study
Source: PLoS One. 2024 Dec 5;19(12):e0315136. doi: 10.1371/journal.pone.0315136 (PMC11620627; doi:10.1371/journal.pone.0315136)
Supplement: Attachment — Submitted filename: Response to Reviewers.docx [file pone.0315136.s004.docx]

**Our response to the reviewers**

Dear reviewers,

We have now made the changes that reviewer 2 suggested by changing the colours of the datapoints so that it is easier to see which data point belongs to the two different treatments, high and low wave level. The figure text has been changed to explain this. We have also made it clear in the figure text that each data point represents an individual mussel.

Regards, Trine
